# Evaluation of Harmonic Contributions for Multi Harmonic Sources System Based on Mixed Entropy Screening and an Improved Independent Component Analysis Method

**DOI:** 10.3390/e22030323

**Published:** 2020-03-12

**Authors:** Jinshuai Zhao, Honggeng Yang, Xiaoyang Ma, Fangwei Xu

**Affiliations:** The College of Electrical Engineering, Sichuan University, Chengdu 610065, China; 2017323030005@stu.scu.edu.cn (J.Z.); pqlab_99@126.com (H.Y.); xufangwei__scu@163.com (F.X.)

**Keywords:** harmonic contribution, multiple harmonic sources, asynchronous measurement, complex independent component analysis, mixed entropy screening mechanism

## Abstract

Evaluating the harmonic contributions of each nonlinear customer is important for harmonic mitigation in a power system with diverse and complex harmonic sources. The existing evaluation methods have two shortcomings: (1) the calculation accuracy is easily affected by background harmonics fluctuation; and (2) they rely on Global Positioning System (GPS) measurements, which is not economic when widely applied. In this paper, based on the properties of asynchronous measurements, we propose a model for evaluating harmonic contributions without GPS technology. In addition, based on the Gaussianity of the measured harmonic data, a mixed entropy screening mechanism is proposed to assess the fluctuation degree of the background harmonics for each data segment. Only the segments with relatively stable background harmonics are chosen for calculation, which reduces the impacts of the background harmonics in a certain degree. Additionally, complex independent component analysis, as a potential method to this field, is improved in this paper. During the calculation process, the sparseness of the mixed matrix in this method is used to reduce the optimization dimension and enhance the evaluation accuracy. The validity and the effectiveness of the proposed methods are verified through simulations and field case studies.

## 1. Introduction

Harmonic sources in a power system become complex and diverse as more nonlinear customers connected to the power grid [1,2]. For a bus with serious harmonic distortion, evaluating the contribution of each customer is essential to identify the dominant harmonic source and to design the effective scheme for harmonic pollution mitigation [3].

**Figure 1:**
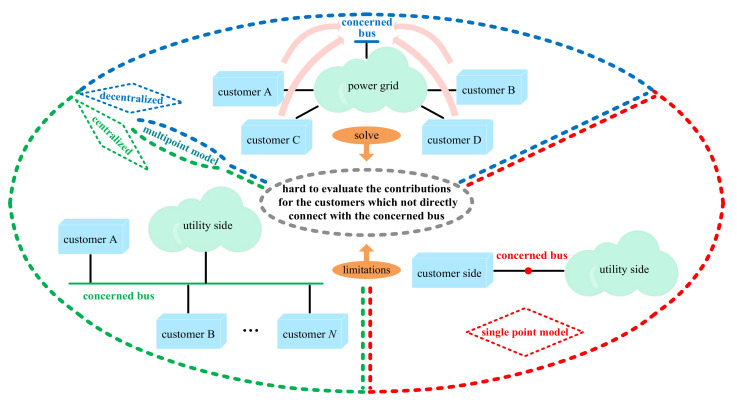
Models for evaluating harmonic contributions.

In general, the evaluation models are classified into two categories (Figure 1): single point and multipoint models. In the single point model, the power grid is divided into the utility and the customer sides at the point of common coupling (PCC), indicating that there is only one suspicious harmonic source. The typical methods to solve this model include the fluctuation method [4], a serious of regression methods [5] and covariance method [6]. Yet, one common limitation of these methods is that they are only suitable when the background harmonics are stable. To reduce the impacts of the background harmonics, from 2015, independent component analysis method (ICA) [7,8] was applied to this researching field [9,10,11,12]. Compared with the classical methods, ICA has a higher calculation accuracy even when the background harmonics fluctuate. Thus, it becomes a popular technology to solve the single point model.

Although the single point model has been widely studied, it is suitable only when a nonlinear customer is directly connected at the concerned bus. However, in practice, harmonic distortion of a bus is usually caused by amplification of harmonics generated by a non-adjacent customer [13]; thus, the single point model is inapplicable in these cases. Furthermore, the wide use of cables in a power grid can potentially increase the risk of harmonic amplification [14,15], which further limits the application of this model. The above limitations have led to research on the multipoint model. This model contains centralized [16,17] and decentralized [18,19,20,21] categories as shown in Figure 1. Nonlinear customers in the centralized model are directly connected at the concerned bus (the PCC). Thus, for a certain customer, the other harmonic sources can be classified into the utility side. As a result, the circuit model can be equivalent to the single point model with similar limitations. In comparison, the decentralized model allows customers to not be directly connected at the concerned bus; thus, it can describe the problem of harmonic contribution more comprehensively.

Compared with the single point model, the developments of the decentralized multipoint model are relatively late. In 2009, a widely accepted index quantifying the harmonic contributions of each nonlinear customer on a concerned bus was proposed by Xu [18]. The idea of this index is that the harmonic contribution of the *i*-th harmonic source can be reflected by the projection of ${\dot{U}}_{i}$ on ${\dot{U}}_{X}$, where ${\dot{U}}_{i}$ denotes the harmonic voltage generated by the *i*-th harmonic source on the concerned bus, while ${\dot{U}}_{X}$ is the harmonic voltage measured at the concerned bus. Based on this index, several methods were proposed to solve the decentralized multipoint model.

**Figure 2:**
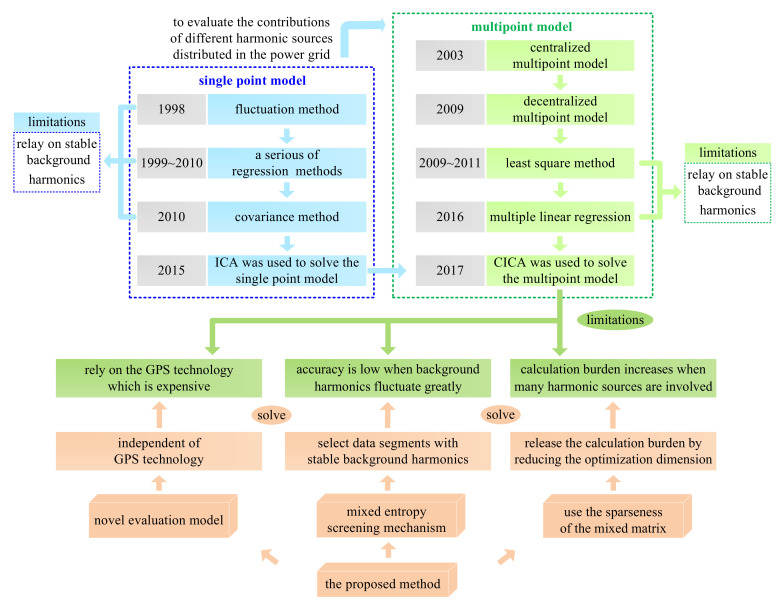
Developments and limitations of the existing methods.

In other studies [18,19], a least squares method was proposed based on the assumptions that the background harmonics are stable and that only one harmonic source fluctuates at a time segment. Although the least squares method is one of the classical methods for harmonic contributions evaluation, in practice, its necessary assumptions are hard to hold for two reasons: (1) a large number of complex nonlinear customers exists in the modern power system, which increases the fluctuation of background harmonics, and (2) it is hard to ensure only one harmonic source fluctuates at a time segment when multiple suspicious harmonic sources are involved. Thus, the application scope of the least squares method is limited. To make the method effective when multiple harmonic sources fluctuate simultaneously, a multiple linear regression method was proposed by Wang and colleagues [20]. This method assumes that for each harmonic source, the phases between ${\dot{U}}_{i}$ and ${\dot{U}}_{X}$ are approximately constant. However, since ${\dot{U}}_{X}$ is generally generated by several nonlinear customers in a multi harmonic sources system, this assumption is hard to hold. Moreover, during the execution of the multiple linear regression method, the background harmonics are still required to be stable. Thus, it has the same limitations as the least squares method. To ensure evaluation accuracy even when background harmonics fluctuate, Wang and co-workers [21] expanded the application of the complex independent component analysis method (CICA) from the single point model to the decentralized multipoint model. Compared with the former two methods, CICA has a superior ability to resist the impacts from the background harmonics. Yet, the calculation results are still unsatisfactory when the background harmonics fluctuate greatly. In addition, the essence of CICA is to reconstruct the source signals based on a negative entropy maximization algorithm. When many suspicious harmonic sources are involved, the calculation burden may increase, and the evaluation accuracy is thus decreased. Furthermore, the CICA method and the evaluation referred to by Wang and co-workers [21] rely on measurements with a Global Positioning System (GPS)-synchronized function that can synchronize the measuring time [18,19,20,21,22,23,24]. However, in engineering practice, it is not realistic to install GPS-based measurements at all related buses because it is uneconomical [6]. Owing to the above three limitations, the existing CICA-based evaluation method still has room for improvements. An overview of the developments and the limitations of the existing methods are shown in Figure 2.

To overcome the shortcomings of the existing CICA method and further accurately evaluate the harmonic contribution for each harmonic source, an improved CICA method is proposed in this paper. First, the properties of asynchronous measurements are explored, and a model independent of GPS is proposed for evaluating harmonic contributions. Then, according to the Gaussianity of the measured harmonic data, a mixed entropy screening mechanism is proposed to select the data segments where the background harmonics are relatively stable. Finally, based on the sparseness of the mixed matrix in the blind source separation model, the optimization dimensions for maximizing the negative entropy in CICA are remarkably decreased. Thus, the calculation burden is significantly reduced, which enhances the evaluation accuracy of the harmonic contributions. Compared with the least squares and multiple linear regression methods, the corresponding assumptions are no longer needed in the proposed method. Meanwhile, as shown in Figure 2, the improved method has three advantages compared with the traditional CICA: (1) it does not rely on GPS-based measurements; (2) it is able to select data segments with stable background harmonics; and (3) it ensures a high calculation accuracy even when the background harmonics fluctuate and/or the number of suspicious harmonics sources is large. The performance of the proposed method is validated by simulations on the IEEE 14-bus system and field case studies with actual multi-infeed high voltage direct current (HVDC) system. For the convenience of reading and understanding, symbols involved in this paper are described in Table 1.

## 2. Decentralized Multipoint Model for Evaluating Harmonic Contributions

### 2.1. Model with Synchronized Phasor Measurements

**Figure 3:**
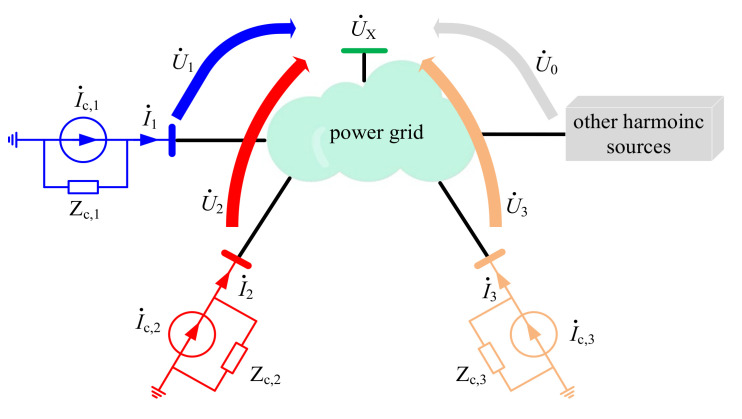
Harmonic model of the concerned bus.

The task of the multipoint model is to evaluate the contribution for each harmonic source at the concerned bus as presented in Figure 3, where ${\dot{U}}_{X}$ is the harmonic voltages measured at the concerned bus, ${\dot{U}}_{0}$ is the background harmonic voltages, ${\dot{U}}_{i}\;\left(i=1,2,3\right)$ is the harmonic voltages generated by the customer *i* at the concerned bus, ${\dot{I}}_{c,i}$ is the harmonic currents generated from the customer *i*, Z_c,*i*_ is the harmonic impedance of customer *i*, and ${\dot{I}}_{i}$ is the harmonic currents measured at the PCC of customer *i*. Among the above harmonic parameters, only ${\dot{U}}_{X}$ and ${\dot{I}}_{i}$ can be measured directly, while other parameters are unknown. Notably, the relative phases between ${\dot{U}}_{X}$ and each ${\dot{I}}_{i}$ are obtained from the GPS-based synchronized phasor measurements.

**Figure 4:**
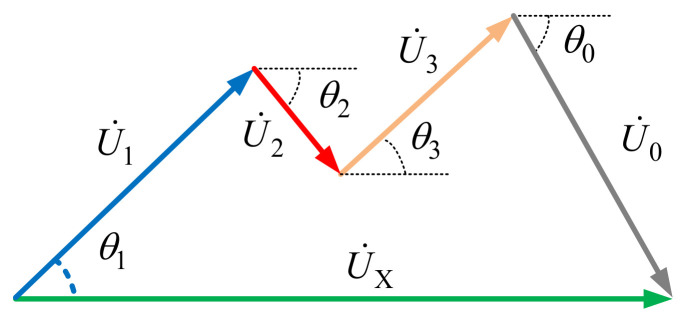
Harmonic voltage at bus X.

In the decentralized multipoint model of Figure 3, the *h*-th harmonic voltages at the concerned bus (${\dot{U}}_{X}$) are generated by all the harmonic sources in the power grid as shown in Figure 4 and Equation (1).

(1) $$\left\{\begin{array}{c} {\dot{U}}_{X}={\dot{U}}_{0}+\sum_{i=1}^{N-1}{\dot{U}}_{i}\\ {\dot{U}}_{i}={\dot{I}}_{c,i}Z_{X,i} \end{array}\right.,$$

where Z_X,*i*_ is the harmonic transfer impedance between customer *i* and bus X, and *N* − 1 is the number of suspicious harmonic sources. 

By projecting ${\dot{U}}_{i}$ onto ${\dot{U}}_{X}$, we obtain the harmonic contribution for customer *i* as:

(2) $$\sigma_{X,i}=\frac{\left|{\dot{U}}_{i}\right|\cdot \cos\left(\theta_{i}\right)}{\left|{\dot{U}}_{X}\right|}\times 100\%.$$

Thus, accurately calculating the transfer impedance Z_X,*i*_ is the key to evaluating the harmonic contribution for each customer.

**Figure 5:**
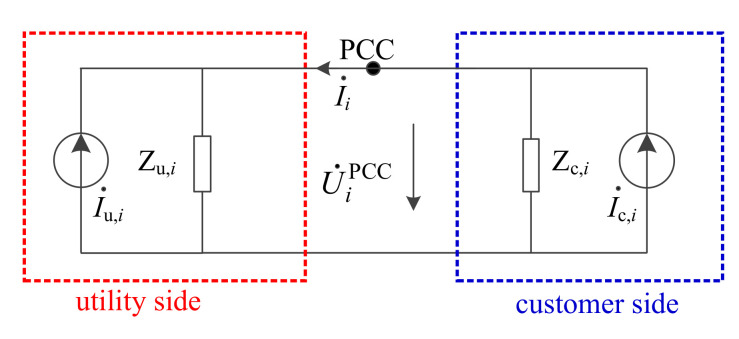
Circuit of the single point model for customer *i*.

To calculate Z_X,*i*_, the first step is to estimate the harmonic currents ${\dot{I}}_{c,i}$ generated from each suspicious harmonic source [21]. This can be done by solving the single point model for each customer. The equivalent harmonic circuit of customer *i* is shown in Figure 5, where the harmonic voltages ${\dot{U}}_{i}^{\mathrm{PCC}}$ and currents ${\dot{I}}_{i}$ can be measured directly at the PCC. ${\dot{I}}_{u,i}$ and Z_u,*i*_ are respectively the harmonic current and impedance of the utility side. 

According to Figure 5, we have

(3) $$\left[\begin{array}{c} {\dot{U}}_{i}^{\mathrm{pcc}}\\ {\dot{I}}_{i} \end{array}\right]=\left[\begin{array}{cc} \frac{Z_{u,i}Z_{c,i}}{Z_{u,i}+Z_{c,i}} & \frac{Z_{u,i}Z_{c,i}}{Z_{u,i}+Z_{c,i}}\\ -\frac{Z_{u,i}}{Z_{u,i}+Z_{c,i}} & \frac{Z_{c,i}}{Z_{u,i}+Z_{c,i}} \end{array}\right]\left[\begin{array}{c} {\dot{I}}_{u,i}\\ {\dot{I}}_{c,i} \end{array}\right],$$

Generally, the fast-varying components of ${\dot{I}}_{c,i}$ and ${\dot{I}}_{u,i}$ separated from an average filter are approximately independent [9,10,11,21]. Thus, the CICA method can be adopted to solve Equation (3), and then ${\dot{I}}_{c,i}$ is obtained (the solving processes were introduced in [9,10] in detail.; to avoid repetition, the specific calculation steps are omitted here). After the harmonic currents ${\dot{I}}_{c,i}$ are generated from each harmonic source estimated, we establish the corresponding blind source separation model as [21]:

(4) $$\underset{X}{\underset{︸}{\left[\begin{array}{c} {\dot{U}}_{X}\\ {\dot{I}}_{c,1}\\ {\dot{I}}_{c,2}\\ \vdots \\ {\dot{I}}_{N-1} \end{array}\right]}}=\underset{A}{\underset{︸}{\left[\begin{array}{ccccc} Z_{X,1} & Z_{X,2} & \cdots & Z_{X,N-1} & 1\\ 1 & 0 & \cdots & 0 & 0\\ 0 & 1 & 0 & \cdots & 0\\ \vdots & \vdots & \ddots & \vdots & \vdots \\ 0 & 0 & \cdots & 1 & 0 \end{array}\right]}}\underset{S}{\underset{︸}{\left[\begin{array}{c} {\dot{I}}_{c,1}\\ {\dot{I}}_{c,2}\\ \vdots \\ {\dot{I}}_{N-1}\\ {\dot{U}}_{0} \end{array}\right]}},$$

Since the fast-varying components among each ${\dot{I}}_{c,i}$ and ${\dot{U}}_{0}$ are approximately independent, Equation (4) can also be solved by the CICA method [21] as

(5) $$\underset{X}{\underset{︸}{\left[\begin{array}{c} {\dot{U}}_{X}^{\mathrm{fast}}\\ {\dot{I}}_{c,1}^{\mathrm{fast}}\\ {\dot{I}}_{c,2}^{\mathrm{fast}}\\ \vdots \\ {\dot{I}}_{c,N-1}^{\mathrm{fast}} \end{array}\right]}}=\underset{\hat{A}}{\underset{︸}{\left[\begin{array}{ccccc} k_{1}{\hat{Z}}_{X,1} & k_{2}{\hat{Z}}_{X,2} & \cdots & k_{N-1}{\hat{Z}}_{X,N-1} & 1\\ k_{1} & 0 & \cdots & 0 & 0\\ 0 & k_{2} & 0 & \cdots & 0\\ \vdots & \vdots & \ddots & \vdots & \vdots \\ 0 & 0 & \cdots & k_{N-1} & 0 \end{array}\right]}}\underset{Y}{\underset{︸}{\left[\begin{array}{c} {\hat{\dot{I}}}_{c,1}^{\mathrm{fast}}\\ {\hat{\dot{I}}}_{c,2}^{\mathrm{fast}}\\ \vdots \\ {\hat{\dot{I}}}_{c,N-1}^{\mathrm{fast}}\\ {\hat{\dot{U}}}_{0}^{\mathrm{fast}} \end{array}\right]}}$$

where the superscript “^fast^” represents the fast-varying components of each signal; the separated signals $Y={\left[\begin{array}{ccccc} {\hat{\dot{I}}}_{c,1}^{\mathrm{fast}} & {\hat{\dot{I}}}_{c,2}^{\mathrm{fast}} & \cdots & {\hat{\dot{I}}}_{c,N-1}^{\mathrm{fast}} & {\hat{\dot{U}}}_{0}^{\mathrm{fast}} \end{array}\right]}^{T}$ correspond to the source signals ${\left[\begin{array}{ccccc} {\dot{I}}_{c,1}^{\mathrm{fast}} & {\dot{I}}_{c,2}^{\mathrm{fast}} & \cdots & {\dot{I}}_{c,N-1}^{\mathrm{fast}} & {\dot{U}}_{0}^{\mathrm{fast}} \end{array}\right]}^{T}$ with scaling indeterminacy [9,10,11,21]. This indeterminacy is indicated by the unknown complex coefficients *k_i_*. The transfer impedances Z_X,*i*_ can be obtained as:

(6) $${\hat{Z}}_{X,i}={\hat{A}}_{1,i}/{\hat{A}}_{i+1,i},$$

where ${\hat{A}}_{i+1,i}$ denotes the element that corresponds to the (*i* + 1)-st row and *i*-th column of matrix $\hat{A}$. 

Based on the calculated ${\hat{Z}}_{X,i}$ and Equation (4), we can recover the matrix $\hat{A}$ that does not contain *k_i_*, and thus, the scaling indeterminacy of the separated signals ***Y*** is solved. Finally, on the basis of the obtained ${\hat{Z}}_{X,i}$ and ${\hat{\dot{U}}}_{0}$, the harmonic contribution for each customer can be assessed by using Equations (1) and (2).

### 2.2. Model with Asynchronized Phasor Measurements

The above model is largely based on GPS-based synchronized phasor measurements, which is not practical as previously stated [6]. As a result, in many cases it is hard to obtain the relative phases between ${\dot{U}}_{X}$ and each ${\dot{I}}_{i}$ in Equation (1). To solve this problem, we propose a novel evaluation model in this section.

#### 2.2.1. Properties of Asynchronous Measurements

Under conditions without GPS technology, a tiny and unknown time difference Δ*t* exists between the starting times of two measurements. For instance, the voltage waves measured at bus X satisfy

(7) $$U_{X}\left(t\right)=U_{X0}+\sum_{h=1}^{\infty }H_{Xh}\cos\left(h\omega_{1}t+\phi_{Xh}\right),$$

where *U*_x0_ is the magnitude of the direct current (DC) component; *H*_x*h*_ and $\phi_{Xh}$ are the magnitude and initial angle of the *h*-th harmonic, respectively; and $\omega_{1}=2\pi f_{1}$ where *f*_1_ is the fundamental frequency.

Assuming the starting time of measurement at bus A is Δ*t* behind that of measurement at bus X, we have

(8) $$U_{A}\left(t\right)=U_{A0}+\sum_{h=1}^{\infty }H_{Ah}\cos\left[h\omega_{1}\left(t+\Delta t\right)+\phi_{Ah}\right],$$

Equation (8) can also be transformed into

(9) $$\left\{\begin{array}{c} U_{A}\left(t\right)=U_{A0}+\sum_{h=1}^{\infty }H_{Ah}\cos\left[h\omega_{1}t+\left(\phi_{Ah}+\alpha_{A}\right)\right]\\ \alpha_{A}=h\omega_{1}\Delta t \end{array}\right.,$$

Adopting the discrete Fourier transform (DFT) for *U*_A_(*t*), we obtain the amplitude and initial angle for the *h*-th harmonic as *H*_A*h*_ and $\left(\phi_{Ah}+\alpha_{A}\right)$. Additionally, if the starting time of measurement at bus A is advanced by Δ*t*, the measurements at buses A and X will be synchronized, and thus, the corresponding DFT results become *H*_A*h*_ and $\phi_{Ah}$. Therefore, two conclusions can be drawn theoretically for the *h*-th harmonic obtained from the DFT:

- The amplitudes are the same for the synchronous and asynchronous measurements.
- The initial angular difference between the synchronous and asynchronous measurements is $h\omega_{1}\Delta t$ (Δt is unknown).

In practice, all of the measuring data are usually divided into several segments to detect the harmonics. Since Δ*t* is the same for each segment, the angular difference between the synchronous and asynchronous measurements is constant for each data segment.

**Figure 6:**
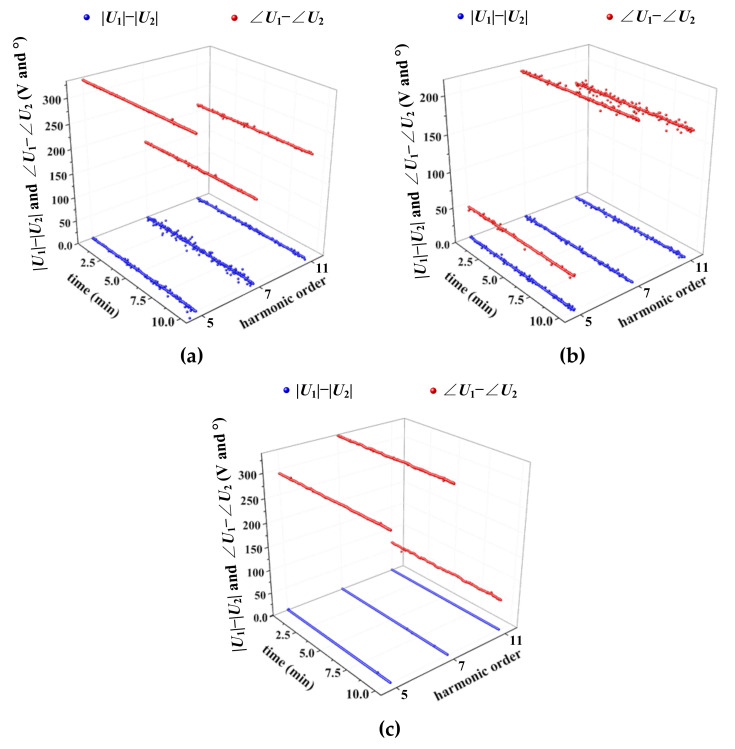
Amplitude and phase analysis of three practice cases: (**a**) wind farm; (**b**) photovoltaic station; and (**c**) nonlinear loads with computers and light-emitting diode (LED) lights.

We further verify the properties of asynchronous measurements for three practice cases: (1) a wind farm; (2) a photovoltaic station; and (3) nonlinear loads with computers and light-emitting diode (LED) lights. The harmonic voltages (5th, 7th, and 11th) obtained from the DFT transformation for each time segment (0.02 s) are defined as ***U*_1_**. After manually creating a measurement delay, we define the corresponding DFT results as ***U*_2_**. Figure 6 presents the difference between ***U*_1_** and ***U*_2_**. For each case, |***U*_1_**|−|***U*_2_**|=0 and the differences between ∠***U*_1_** and ∠***U*_2_** are constant; thus, the aforementioned two properties are valid. Consequently, the amplitudes of harmonics detected from synchronous and asynchronous data are the same, while their phase differences are constant.

#### 2.2.2. Proposed Model for Evaluating Harmonic Contributions Without GPS Technology

On the basis of the analysis above, we can transform Equation (4) by setting the starting time of measurement at bus X as the reference:

(10) $$\left[\begin{array}{c} {\dot{U}}_{X}\\ e^{j\alpha_{1}}{\dot{I}}_{c,1}\\ e^{j\alpha_{2}}{\dot{I}}_{c,2}\\ \vdots \\ e^{j\alpha_{N-1}}{\dot{I}}_{c,N-1} \end{array}\right]=\left[\begin{array}{ccccc} Z_{X,1} & Z_{X,2} & \cdots & Z_{X,N-1} & 1\\ e^{j\alpha_{1}} & 0 & \cdots & 0 & 0\\ 0 & e^{j\alpha_{2}} & 0 & \cdots & 0\\ \vdots & \vdots & \ddots & \vdots & \vdots \\ 0 & 0 & \cdots & e^{j\alpha_{N-1}} & 0 \end{array}\right]\left[\begin{array}{c} {\dot{I}}_{c,1}\\ {\dot{I}}_{c,2}\\ \vdots \\ {\dot{I}}_{c,N-1}\\ {\dot{U}}_{0} \end{array}\right],$$

where $\alpha_{i}\left(i=1,2,\cdots ,N-1\right)$ represents the relative phase between the synchronous and asynchronous measurements for customers *i*. Notably, the source signal ${\dot{I}}_{c,i}$ is still synchronous with ${\dot{U}}_{X}$. Yet, because $\alpha_{i}$ is unknown, we only have the asynchronous signals $e^{j\alpha_{i}}{\dot{I}}_{c,i}$.

Since the fluctuations of ${\dot{I}}_{c,i}$ and ${\dot{u}}_{0}$ are independent from each other [9,10,11,21], Equation (10) can be solved via CICA as:

(11) $$\underset{X}{\underset{︸}{\left[\begin{array}{c} {\dot{U}}_{X}^{\mathrm{fast}}\\ {\left(e^{j\alpha_{1}}{\dot{I}}_{c,1}\right)}^{\mathrm{fast}}\\ {\left(e^{j\alpha_{2}}{\dot{I}}_{c,2}\right)}^{\mathrm{fast}}\\ \vdots \\ {\left(e^{j\alpha_{N-1}}{\dot{I}}_{c,N-1}\right)}^{\mathrm{fast}} \end{array}\right]}}=\underset{\hat{A}}{\underset{︸}{\left[\begin{array}{ccccc} k_{1}{\hat{Z}}_{X,1} & k_{2}{\hat{Z}}_{X,2} & \cdots & k_{N-1}{\hat{Z}}_{X,N-1} & 1\\ k_{1}e^{j\alpha_{1}} & 0 & \cdots & 0 & 0\\ 0 & k_{2}e^{j\alpha_{2}} & 0 & \cdots & 0\\ \vdots & \vdots & \ddots & \vdots & \vdots \\ 0 & 0 & \cdots & k_{N-1}e^{j\alpha_{N-1}} & 0 \end{array}\right]}}\underset{Y}{\underset{︸}{\left[\begin{array}{c} {\hat{\dot{I}}}_{c,1}^{\mathrm{fast}}\\ {\hat{\dot{I}}}_{c,2}^{\mathrm{fast}}\\ \vdots \\ {\hat{\dot{I}}}_{c,N-1}^{\mathrm{fast}}\\ {\hat{\dot{U}}}_{0}^{\mathrm{fast}} \end{array}\right]}}$$

where the unknown complex coefficients *k_i_* indicate the scaling indeterminacy [9,10,11,21].

The transfer impedances Z_X,*i*_ combined with α*_i_* can be obtained as:

(12) $$e^{-j\alpha_{i}}{\hat{Z}}_{X,i}={\hat{A}}_{1,i}/{\hat{A}}_{i+1,i},$$

However, we still cannot separate the transfer impedances from α*_i_* because these angles are unknown. Yet, according to Equations (1) and (12), we can evaluate the harmonic contributions via $e^{-j\alpha_{i}}{\hat{Z}}_{X,i}$ without separating the transfer impedances from these unknown angles. When signal $e^{j\alpha_{i}}{\hat{\dot{I}}}_{c,i}$ multiplies $e^{-j\alpha_{i}}$, the new signal ${\hat{\dot{I}}}_{c,i}$ is synchronized with ${\dot{U}}_{X}$. Although α*_i_* is unknown, based on Equations (1) and (12), we have

(13) $$\left(e^{j\alpha_{i}}{\hat{\dot{I}}}_{c,i}\right)\left(e^{-j\alpha_{i}}{\hat{Z}}_{X,i}\right)={\hat{\dot{I}}}_{c,i}{\hat{Z}}_{X,i}={\hat{\dot{U}}}_{i}.$$

Thus, ${\hat{\dot{I}}}_{c,i}{\hat{Z}}_{X,i}$ is obtained directly without calculating α*_i_* because the angle α*_i_* in $e^{j\alpha_{i}}{\hat{\dot{I}}}_{c,i}$ and $e^{-j\alpha_{i}}{\hat{Z}}_{X,i}$ is just offset. Consequently, the harmonic contribution of each source can be evaluated without the synchronized phasor measurements.

## 3. A Mixed Entropy Screening Mechanism for Data Segments Selection

During the calculation process of CICA, the harmonic impedances should be constant to keep the matrix ***A*** invariant. To ensure this, first, the whole measured data are usually divided into several short segments. Then, we calculate the transfer impedances using each segment separately and average the results. It can be considered that the harmonic impedances are approximately constant during each single data segment because the corresponding time is quite short. Additionally, the calculation accuracy of CICA relies on stable background harmonics. A large fluctuation of the background harmonics may increase the calculation errors [9,10,11,21]. Therefore, if the fluctuation degree of the background harmonics is assessable, we can improve the calculation accuracy by choosing the data segments with relatively stable background harmonics for calculation, while eliminating the data segments with heavily fluctuating background harmonics. The problem is that the background harmonics cannot be measured directly. To overcome this difficulty, a mixed entropy screening mechanism is proposed in this section.

**Figure 7:**
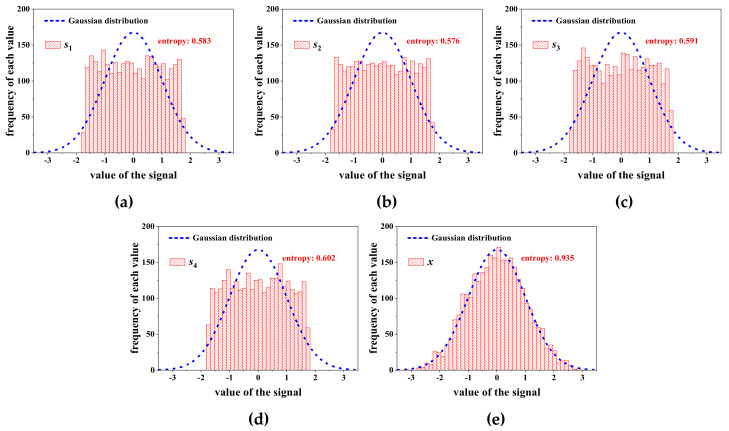
Distribution and entropy of each signal: (**a**) *s*_1_; (**b**) *s*_2_; (**c**) *s*_3_; (**d**) *s*_4_; (**e**) *x*.

Based on the central limit theorem [25], the distribution of a signal *x* linear combined by many random signals tends to be Gaussian. In addition, if signal *x* is only dominated by a few of these signals, its Gaussian degree will be decreased. For instance, signal *x* is combined by four random real signals $s_{i}\;\;\left(i=1,2,3,4\right)$ with unit amplitude as: $X=s_{1}+s_{2}+s_{3}+s_{4}$. The sample size for each signal is 3000. Their distributions and Gaussian degrees are shown in Figure 7, where the Gaussian degree is assessed by the entropy of a signal as Equation (14) [26]. A high entropy corresponds to a strong Gaussian degree.

(14) $$H\left(s\right)=-\sum_{m=1}^{M}p\left(s_{m}\right)\log_{2}p\left(s_{m}\right),$$

where *M* is the sample size of signal *s*, $p\left(s_{m}\right)$ is the probability mass function of *s*.

Since the standard Gaussian signal has the largest entropy among all random signals of equal variance [27], to make the entropy of different signals comparable, transformations in Equations (15) and (16) are performed for each signal.

(15) $$\left\{\begin{array}{c} s\;\;\to \;\;s-E\left\{s\right\}\\ s\to \;\;s/std\left\{s\right\} \end{array}\right.,$$

where $E\left\{s\right\}$ and $std\left\{s\right\}$ are the mean value and standard deviation of signal *s*, respectively.

(16) $$H\left(s\right)\;\;\to \;\;H\left(s\right)/H\left(s_{G}\right),$$

where $s_{G}$ is the standard Gaussian signal with zero-mean and unit variance.

**Figure 8:**
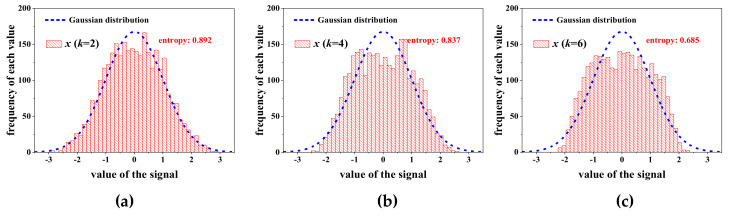
Distribution and entropy of the mix signal *x* in each case: (**a**) *k* = 2; (**b**) *k* = 4; (**c**) *k* = 6.

As shown in Figure 7, compared with the signals $s_{i}\;\left(i=1,2,3,4\right)$, the mixed signal *x* is closer to Gaussian distribution. Meanwhile, the entropy of signal *x* is also the biggest one among these signals, revealing that entropy can correctly reflect the Gaussian degree of each signal. After the amplitude of signal *s*_4_ increases *k* times, the distribution and the entropy of signal *x* is presented in Figure 8. With the increasing of *k*, signal *x* is gradually dominated by *s*_4_, which causes the decline of the Gaussian degree of *x*, and so, the corresponding entropy declines. 

The above analyses can be applied into Equation (10) to assess the fluctuation degree of the background harmonic voltages ${\dot{U}}_{0}$. Since the Gaussian degree of each complex signal in Equation (10) can be reflected by the Gaussian degree of their real and imaginary parts [11], we use the mixed entropy defined in Equation (17) to assess the Gaussian degree of a complex signal. For a data segment, two possible situations are discussed below:

(17) $$H\left[s\right]=\frac{H\left(s_{X}\right)+H\left(s_{y}\right)}{2},$$

where the subscripts “_x_” and “_y_” respectively represent the real and imaginary parts of signal *s*.

- Case (1): The background harmonics fluctuate greatly:

In this case, ${\dot{U}}_{X}^{\mathrm{fast}}$ is dominated by the fast-varying components of the background harmonics ${\dot{U}}_{0}^{\mathrm{fast}}$, and thus, the Gaussian degree of ${\dot{U}}_{X}^{\mathrm{fast}}$ becomes relatively low. The mixed entropy of ${\dot{U}}_{X}^{\mathrm{fast}}$ approaches $H\left[{\dot{U}}_{0}^{\mathrm{fast}}\right]$ and is small compared with the data segment where ${\dot{U}}_{0}$ is stable.

- Case (2): The background harmonics are stable:

In this case, ${\dot{U}}_{X}^{\mathrm{fast}}$ usually has a relatively high Gaussian degree. However, if one of the harmonic sources fluctuates greatly in this data segment, ${\dot{U}}_{X}^{\mathrm{fast}}$ will be dominated by ${\dot{I}}_{c,i}^{\mathrm{fast}}$. Thus, the mixed entropy of ${\dot{U}}_{X}^{\mathrm{fast}}$ approaches $H\left[{\dot{I}}_{c,i}^{\mathrm{fast}}\right]$ and is still small compared with the cases where ${\dot{I}}_{c,i}$ is relatively stable. Yet, this case is still quite different from case (1). Generally, the background harmonic voltages are the combination of other harmonics in the power grid. Thus, compared with the current signal ${\dot{I}}_{c,i}$ generated from a single nonlinear customer, the Gaussian degree of ${\dot{U}}_{0}^{\mathrm{fast}}$ is usually stronger according to the central limit theorem [11]. Therefore, $H\left[{\dot{U}}_{0}^{\mathrm{fast}}\right]$ is usually larger than $H\left[{\dot{I}}_{c,i}^{\mathrm{fast}}\right]$. 

Although ${\dot{I}}_{c,i}^{\mathrm{fast}}$ cannot be directly obtained under the situation of asynchronous measurements, the distribution of ${\dot{I}}_{c,i}^{\mathrm{fast}}$ and ${\left(e^{j\alpha_{i}}{\dot{I}}_{c,i}\right)}^{\mathrm{fast}}$ are approximately coincident. Thus, for a data segment where $H\left[{\dot{U}}_{X}^{\mathrm{fast}}\right]$ is relatively small, if $H\left[{\dot{U}}_{X}^{\mathrm{fast}}\right]$ is obviously larger than $H\left[{\left(e^{j\alpha_{i}}{\dot{I}}_{c,i}\right)}^{\mathrm{fast}}\right]\;\;\left(i=1,2,\cdots ,N-1\right)$, the background harmonics fluctuate greatly. Otherwise, if $H\left[{\dot{U}}_{X}^{\mathrm{fast}}\right]$ is close to or even smaller than one of the $H\left[{\left(e^{j\alpha_{i}}{\dot{I}}_{c,i}\right)}^{\mathrm{fast}}\right]$, then the background harmonics will be stable. Empirically, when Equation (18) holds, it can be considered that $H\left[{\dot{U}}_{X}^{\mathrm{fast}}\right]$ is close to or smaller than $H\left[{\left(e^{j\alpha_{i}}{\dot{I}}_{c,i}\right)}^{\mathrm{fast}}\right]$.

(18) $$\sigma_{H}=\min\left[\frac{H\left[{\dot{U}}_{X}^{\mathrm{fast}}\right]-H\left[{\left(e^{j\alpha_{i}}{\dot{I}}_{c,i}\right)}^{\mathrm{fast}}\right]\;}{H\left[{\left(e^{j\alpha_{i}}{\dot{I}}_{c,i}\right)}^{\mathrm{fast}}\right]}\times 100\%\right]<4\%\;\;\left(i=1,2,\cdots ,N-1\right),$$

For all the given data segments, the mixed entropy for their corresponding signal ${\dot{U}}_{X}^{\mathrm{fast}}$ can be obtained by using Equation (17). Furthermore, the average value of these mixed entropies can be calculated (defined as **H**_m_). If $H\left[{\dot{U}}_{X}^{\mathrm{fast}}\right]>H_{m}$ holds for a data segment, the corresponding background harmonics are stable. Otherwise, Equation (18) is required to further assess the fluctuation degree of the background harmonics. 

**Figure 9:**
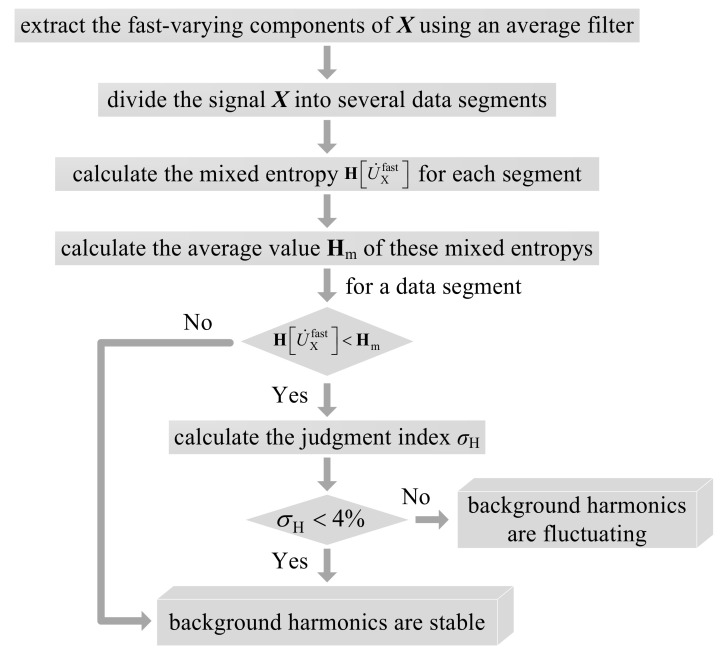
Schematic diagram of the mixed entropy screening mechanism.

A schematic diagram of the mixed entropy screening mechanism is shown in Figure 9.

## 4. Improved CICA Method

Although the mixed entropy screening mechanism can release the impacts of background harmonics, to further improve calculation accuracy, an improved CICA is proposed in this section. Before applying the CICA method, the observed signals ***X*** are preprocessed by centering and whitening to simplify the calculation [9,10,11]. The centering process transforms ***X*** into zero-mean signals, while the whitening process can be done as

(19) $$X_{w}=QX,$$

(20) $$Q=\Lambda^{-0.5}\Gamma^{T},$$

where ***Q*** is the whitening matrix. ***Λ*** and ***Γ*** are the diagonal and orthogonal matrices of eigenvalues of *E*{***XX***^T^}, respectively (the symbol *E*{.} denotes a mean value) [10].

The key step of CICA is to find a separating matrix ***W*** satisfying

(21) $$Y=W^{T}X_{w},$$

where the symbol ^T^ represents the Hermitian transpose.

CICA is essentially an optimization algorithm based on the central limit theorem. Its goal is to maximize the non-Gaussianities of each signal in matrix ***Y***, while the elements in matrix ***W*** are the variables to be optimized [7,8,28]. The non-Gaussianities of signal ***W***^T^***X***_w_ (signal ***Y***) can be estimated via the negative entropy [7,11,29,30]:

(22) $$J_{G}(W^{T}X_{W})\approx {\left[E\left\{G\left(W^{T}X_{W}\right)\right\}-E\left\{G\left(s_{Gauss}\right)\right\}\right]}^{2},$$

where *s*_Gauss_ is a signal with zero mean and unit variance and obeys a Gaussian distribution, and *G*{.} is a nonlinear function. The non-Gaussianity of signal ***W***^T^***X***_w_ strengthens as the negative entropy *J*_G_(***W***^T^***X***_w_) increases.

Of note, the maxima of *J*_G_(***W***^T^***X***_w_) are obtained when $E\left\{G\left(W^{T}X_{W}\right)\right\}$ is optimal [7]. Furthermore, in another study [8], by defining *g*{.} as the derivative of *G*{.}, index $\sigma_{c}$ is deduced to solve this optimization problem:

(23) $$\sigma_{c}=E\left\{g\left({\left|W^{T}X_{W}\right|}^{2}\right)+{\left|W^{T}X_{W}\right|}^{2}g^{\prime }\left({\left|W^{T}X_{W}\right|}^{2}\right)-\right.\;\left.{\left|W^{T}X_{W}\right|}^{2}g\left({\left|W^{T}X_{W}\right|}^{2}\right)\right\}.$$

When $\sigma_{c}<0$, we should calculate the matrix ***W*** that maximizes $E\left\{G\left({\left|W^{T}X_{W}\right|}^{2}\right)\right\}$. Conversely, when $\sigma_{c}>0$, the matrix ***W*** that minimizes $E\left\{G\left({\left|W^{T}X_{W}\right|}^{2}\right)\right\}$ should be solved [8]. The problem of maximizing the negative entropy *J*_G_(***W***^T^***X***_w_) is now converted into optimizing $E\left\{G\left({\left|W^{T}X_{W}\right|}^{2}\right)\right\}$.

Despite the ordering and scaling indeterminacies of CICA [9,10,11,21], the optimal ***W*** satisfies

(24) $$W^{T}=A^{-1}Q^{-1}.$$

Additionally, matrix ***A*** in Equation (10) is sparse to a certain degree. For an *N* × *N* matrix ***A***, there are (*N* − 1) × (*N* − 1) elements naturally equal to 0 and one element equal to 1. However, in traditional CICA, these known elements are still treated as variables to be solved, which may cause two problems: (1) wasting the chance to reduce the optimization dimensions; and (2) increasing errors when the calculation results of these elements do not equal to their theoretical values (0 or 1). 

This paper uses the sparseness of matrix ***A*** to improve CICA and enhance the calculation accuracy. By assuming there are *N* − 1 suspicious harmonic sources in a system, we calculate the inverse of matrix ***A*** as

(25) $$A^{-1}=\left[\begin{array}{ccccc} 0 & e^{-j\alpha_{1}} & 0 & \cdots & 0\\ 0 & 0 & e^{-j\alpha_{2}} & \cdots & 0\\ \vdots & \vdots & \vdots & \ddots & \vdots \\ 0 & 0 & \cdots & 0 & e^{-j\alpha_{N-1}}\\ 1 & -Z_{X,1} & -Z_{X,2} & \cdots & -Z_{X,N-1} \end{array}\right].$$

Since matrix ***Q*** can be obtained from ***X***, we can calculate its inverse ***Q***^−1^ (defined as Θ). Thus, Equation (24) gives

(26) $$W=\left[\begin{array}{ccccc} \Theta_{2,1}e^{-j\alpha_{1}} & \Theta_{3,1}e^{-j\alpha_{2}} & \cdots & \Theta_{N,1}e^{-j\alpha_{N-1}} & W_{1,N}\\ \Theta_{2,2}e^{-j\alpha_{1}} & \Theta_{3,2}e^{-j\alpha_{2}} & \cdots & \Theta_{N,2}e^{-j\alpha_{N-1}} & W_{2,N}\\ \vdots & \vdots & \cdots & \vdots & \vdots \\ \Theta_{2,N}e^{-j\alpha_{1}} & \Theta_{3,N}e^{-j\alpha_{2}} & \cdots & \Theta_{N,N}e^{-j\alpha_{N-1}} & W_{N,N} \end{array}\right],$$

where $W_{N,N}$ and $\Theta_{N,N}$ represent the element in the *N*^th^ row and *N*^th^ column of matrix ***W*** and Θ, respectively.

Equation (26) can be rewritten as

(27) $$W=\left[\begin{array}{cccc} W_{1,1} & \cdots & W_{1,N-1} & W_{1,N}\\ W_{1,1}\frac{\Theta_{2,2}}{\Theta_{2,1}} & \cdots & W_{1,N-1}\frac{\Theta_{N,2}}{\Theta_{N,1}} & W_{2,N}\\ \vdots & \cdots & \vdots & \vdots \\ W_{1,1}\frac{\Theta_{2,N}}{\Theta_{2,1}} & \cdots & W_{1,N-1}\frac{\Theta_{N,N}}{\Theta_{N,1}} & W_{N,N} \end{array}\right].$$

Once, $W_{1,1},W_{1,2},\cdots ,W_{1,N}$ and $W_{2,N},W_{3,N},\cdots ,W_{N,N}$ are solved, the other elements in matrix ***W*** are obtained. 

Furthermore, because

(28) $$\left[\begin{array}{cccc} W_{1,N} & W_{2,N} & \cdots & W_{N,N} \end{array}\right]=\left[\begin{array}{cccc} 1 & -Z_{X,1} & \cdots & -Z_{X,N-1} \end{array}\right]\Theta ,$$

we have

(29) $${\left[\begin{array}{c} -Z_{X,1}\\ -Z_{X,2}\\ \vdots \\ -Z_{X,N-1} \end{array}\right]}^{T}=\left({\left[\begin{array}{c} W_{1,N}\\ W_{2,N}\\ \vdots \\ W_{N-1,N} \end{array}\right]}^{T}-{\left[\begin{array}{c} \Theta_{1,1}\\ \Theta_{1,2}\\ \vdots \\ \Theta_{1,N-1} \end{array}\right]}^{T}\right){\left[\begin{array}{cccc} \Theta_{2,1} & \Theta_{2,2} & \cdots & \Theta_{2,N-1}\\ \Theta_{3,1} & \Theta_{3,2} & \cdots & \Theta_{3,N-1}\\ \vdots & \vdots & \cdots & \vdots \\ \Theta_{N,1} & \Theta_{N,2} & \cdots & \Theta_{N,N-1} \end{array}\right]}^{-1}.$$

Thus, *W_N,N_* can be obtained from $W_{2,N},W_{3,N},\cdots ,W_{N-1,N}$, and Θ as

(30) $$W_{N,N}=\left[\begin{array}{cccc} 1 & -Z_{X,1} & \cdots & -Z_{X,N-1} \end{array}\right]\left[\begin{array}{c} \Theta_{1,N}\\ \Theta_{2,N}\\ \vdots \\ \Theta_{N,N} \end{array}\right].$$

**Figure 10:**
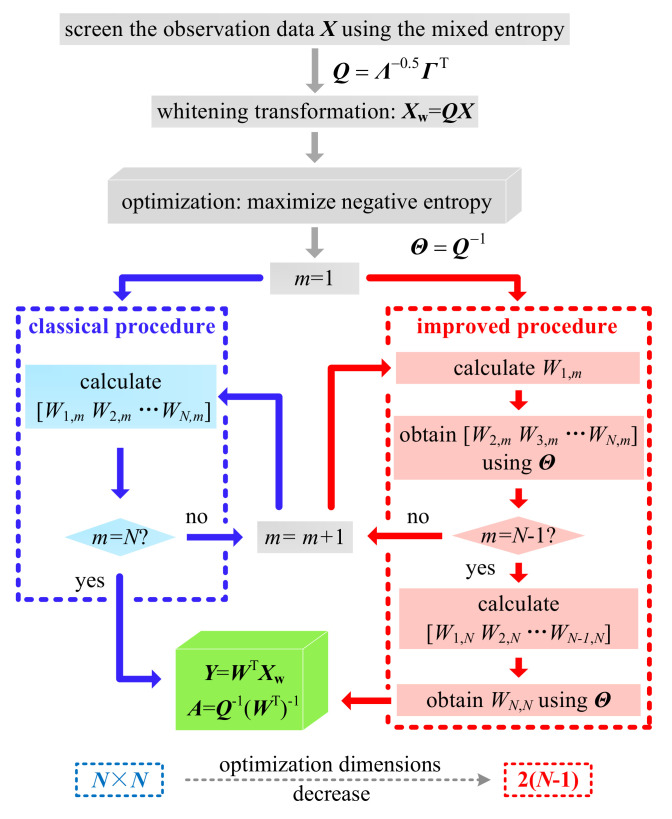
Flow chart of the improved CICA.

Consequently, the optimization dimensions are greatly reduced from *N* × *N* to 2(*N* − 1), which significantly reduces the optimization burden. Moreover, the elements that are already known (0 or 1) in the solved matrix ***A*** will be exactly equal to their proper values without the risk of error. The calculation accuracy is thus further enhanced. The flow chart of the improved CICA is shown in Figure 10.

## 5. Simulation Cases and Analysis

**Figure 11:**
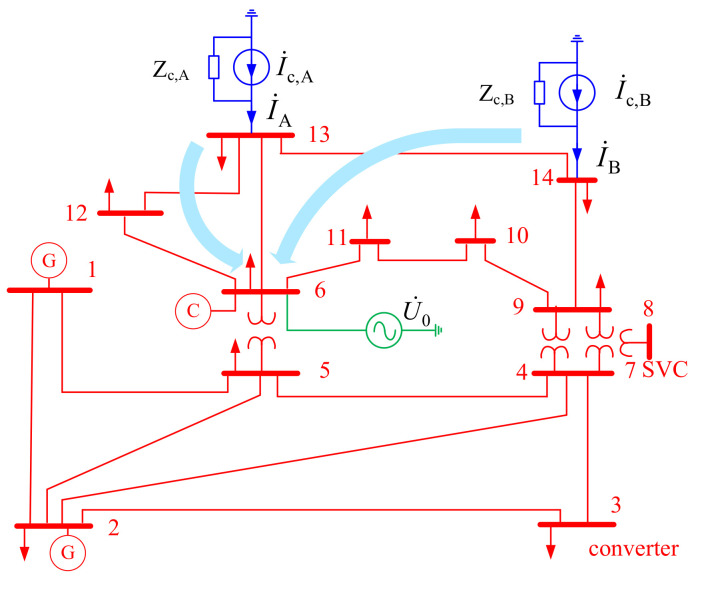
Structure of the IEEE-14 bus system.

**Figure 12:**
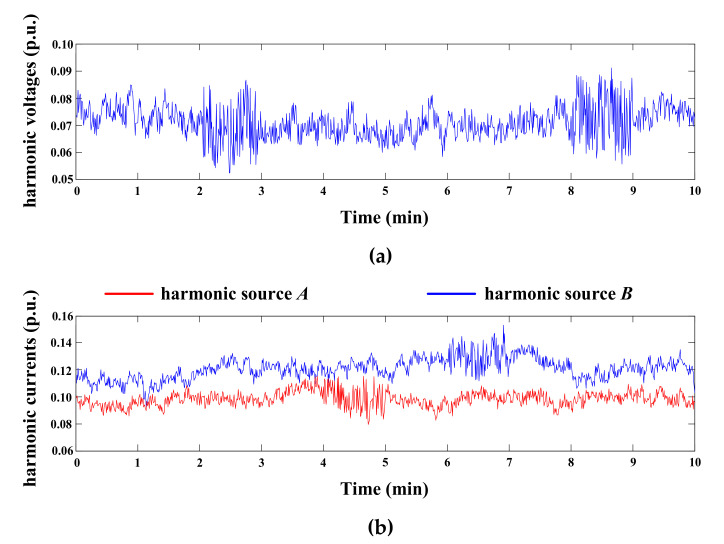
Injected harmonic voltages and currents: (**a**) background harmonic voltages; (**b**) injected harmonic currents of each customer.

The IEEE-14 bus system [31] is used to validate the proposed method. The simulations are processed by the Matlab 8.5 program under the 5th harmonic. Bus 6 is set as the concerned bus, and suspicious harmonic sources are set at buses 13 and 14 (Figure 11). The injected harmonic currents and background harmonic voltages are shown in Figure 12. Please note, all these harmonic data are per unit values, and the corresponding actual values can be converted by their base values in another study [31]. The harmonic data are averaged in 1 second, so we have 1 sample per second. The asynchronously measured harmonic currents are ${\dot{I}}_{c,A}^{\alpha_{A}}=e^{j\alpha_{A}}{\dot{I}}_{c,A}$ and ${\dot{I}}_{c,B}^{\alpha_{B}}=e^{j\alpha_{B}}{\dot{I}}_{c,B}$. Of note, ${\dot{I}}_{c,A}$ and ${\dot{I}}_{c,B}$ are synchronous with the voltages measured at bus 6, while the unknown phases $\alpha_{A}$ and $\alpha_{B}$ are used to simulate asynchronous measurement. During the calculation process, ${\dot{I}}_{c,A}^{\alpha_{A}}$ and ${\dot{I}}_{c,B}^{\alpha_{B}}$ are used to evaluate the harmonic contributions, and the evaluation results from ${\dot{I}}_{c,A}$ and ${\dot{I}}_{c,B}$ are just used as the reference to validate the calculation.

According to the structure and parameters of the IEEE-14 bus system [31], the harmonic impedances of the utility side are $Z_{u,A}=0.1747+0.7313j\;p.u.$ and $Z_{u,B}=0.2708+1.0004j\;p.u.$. Meanwhile, the harmonic impedances of the customer side are set as $Z_{c,A}=0.5+2j\;p.u.$ and $Z_{c,B}=2+11j\;p.u.$. Throughout this simulation, Z_u,A_, Z_u,B_, Z_c,A_, and Z_c,B_ are all unknowns and need to be solved. The above impedance values are just used as the references for analyzing the calculation errors.

### 5.1. Effect of the Mixed Entropy Screening Mechanism

First, to obtain the observation signals ${\left[\begin{array}{ccc} {\dot{U}}_{X} & {\dot{I}}_{c,A}^{\alpha_{A}} & {\dot{I}}_{c,B}^{\alpha_{B}} \end{array}\right]}^{T}$ for Equation (10), the harmonic currents generated by customer *A* and *B* are estimated by solving the single point model in Equation (3) via the CICA method. Then, the fast-varying components ${\left[\begin{array}{ccc} {\dot{U}}_{X}^{\mathrm{fast}} & {\left({\dot{I}}_{c,A}^{\alpha_{A}}\right)}^{\mathrm{fast}} & {\left({\dot{I}}_{c,B}^{\alpha_{B}}\right)}^{\mathrm{fast}} \end{array}\right]}^{T}$ are separated from ${\left[\begin{array}{ccc} {\dot{U}}_{X} & {\dot{I}}_{c,A}^{\alpha_{A}} & {\dot{I}}_{c,B}^{\alpha_{B}} \end{array}\right]}^{T}$ by an average filter. After that, the whole observation signals are divided into 10 segments, and the mixed entropy screening mechanism is adopted to choose the data segments where the background harmonics are relatively stable. 

The calculated mixed entropies of signals ${\dot{U}}_{X}^{\mathrm{fast}}$, ${\left({\dot{I}}_{c,A}^{\alpha_{A}}\right)}^{\mathrm{fast}}$, and ${\left({\dot{I}}_{c,B}^{\alpha_{B}}\right)}^{\mathrm{fast}}$ in each segment are presented in Table 2. Since ${\dot{U}}_{X}^{\mathrm{fast}}$ is mixed by ${\dot{I}}_{c,A}$, ${\dot{I}}_{c,B}^{\mathrm{fast}}$, and ${\dot{U}}_{0}^{\mathrm{fast}}$, according to the central limit theorem, the Gaussianity of ${\dot{U}}_{X}^{\mathrm{fast}}$ is generally the largest among these signals. Table 2 indicates that the mean values of $H\left[{\left({\dot{I}}_{c,A}^{\alpha_{A}}\right)}^{\mathrm{fast}}\right]$ and $H\left[{\left({\dot{I}}_{c,B}^{\alpha_{B}}\right)}^{\mathrm{fast}}\right]$ are obviously smaller than the averages of $H\left[{\dot{U}}_{X}^{\mathrm{fast}}\right]$ (i.e., **H**_m_). Meanwhile, the mean value of $\sigma_{H}$ is positive and far larger than 0. Thus, based on Equation (18), it can be concluded that the Gaussianity of ${\dot{U}}_{X}^{\mathrm{fast}}$ is indeed the strongest in most cases. Theoretically, when the background harmonics fluctuate greatly, ${\dot{U}}_{X}^{\mathrm{fast}}$ is dominated by ${\dot{U}}_{0}^{\mathrm{fast}}$ and so $H\left[{\dot{U}}_{X}^{\mathrm{fast}}\right]$ decreases. Yet, since ${\dot{U}}_{0}^{\mathrm{fast}}$ is also the combination of other harmonic sources, $H\left[{\dot{U}}_{X}^{\mathrm{fast}}\right]$ is still larger than $H\left[{\left({\dot{I}}_{c,A}^{\alpha_{A}}\right)}^{\mathrm{fast}}\right]$ and $H\left[{\left({\dot{I}}_{c,B}^{\alpha_{B}}\right)}^{\mathrm{fast}}\right]$ as analyzed in Section 3. For instance, at the 3rd and 9th segments, the mixed entropy of ${\dot{U}}_{X}^{\mathrm{fast}}$ is relatively lower than **H**_m_ but larger than $H\left[{\left({\dot{I}}_{c,A}^{\alpha_{A}}\right)}^{\mathrm{fast}}\right]$ and $H\left[{\left({\dot{I}}_{c,B}^{\alpha_{B}}\right)}^{\mathrm{fast}}\right]$; thus, it can be considered that ${\dot{U}}_{0}$ fluctuates greatly at these two segments. In comparation, at the 5th and 7th segments, both $H\left[{\dot{U}}_{X}^{\mathrm{fast}}\right]<H_{m}$ and $\sigma_{H}<4\%$ hold, which indicates that $H\left[{\dot{U}}_{X}^{\mathrm{fast}}\right]$ is not only smaller than $H_{m}$, but also close or even smaller than $H\left[{\left({\dot{I}}_{c,A}^{\alpha_{A}}\right)}^{\mathrm{fast}}\right]$ or $H\left[{\left({\dot{I}}_{c,B}^{\alpha_{B}}\right)}^{\mathrm{fast}}\right]$. Therefore, according to the evaluation process in Figure 9, the corresponding background harmonics are stable, while the harmonic currents generated by customer *A* or *B* fluctuate. The above conclusions are just consistent with the curves in Figure 12, and the effect of the mixed entropy screening mechanism is thus verified.

### 5.2. Multipoint Calculation

The key step of multipoint calculation is to solve the transfer impedance between each harmonic source and the concerned bus X (bus 6 in this case). As analyzed before, the calculated transfer impedances without synchronous measurements are $Z_{X,A}^{\alpha_{A}}=e^{-j\alpha_{A}}Z_{X,A}$ and $Z_{X,B}^{\alpha_{B}}=e^{-j\alpha_{B}}Z_{X,B}$. According to Equation (13), the contributions from each harmonic source can be calculated even though $\alpha_{A}$ and $\alpha_{B}$ are unknown.

Four methods are used for calculation: (1) the least squares method [18]; (2) the multiple linear regression method [20]; (3) traditional CICA; and (4) the proposed method. The calculation results are shown in Table 3 and Table 4. For the least squares method, the calculation errors are huge because its necessary assumptions that the background harmonics are stable and that only one harmonic source fluctuates at a time are hard to hold for most of the segments. In addition, in engineering practice, with the increasing number of complex nonlinear customers, several suspicious harmonic sources usually exist for a concerned bus; thus, the basic assumptions of the least squares method are more difficult to satisfy. Meanwhile, the multiple linear regression method also requires that the background harmonics should not fluctuate; thus, the calculation errors are still high. Additionally, the calculation accuracy of traditional CICA is improved but still unsatisfactory because its calculation results are still impacted by the fluctuation of the background harmonics to a certain degree. In comparison, the improved CICA can accurately calculate the transfer impedances and evaluate the harmonic contribution correctly for each harmonic source. 

**Figure 13:**
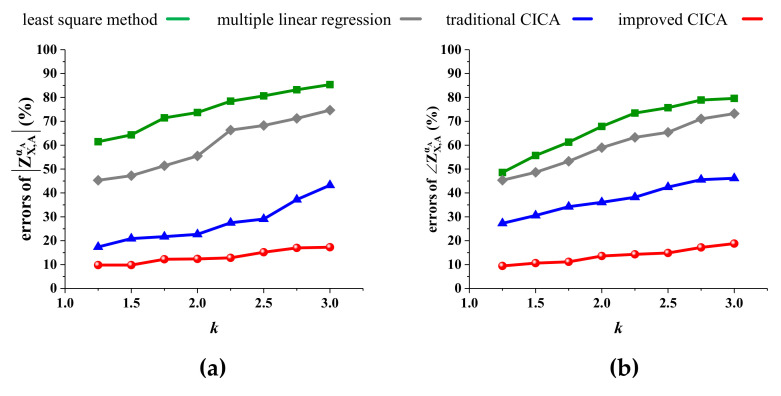

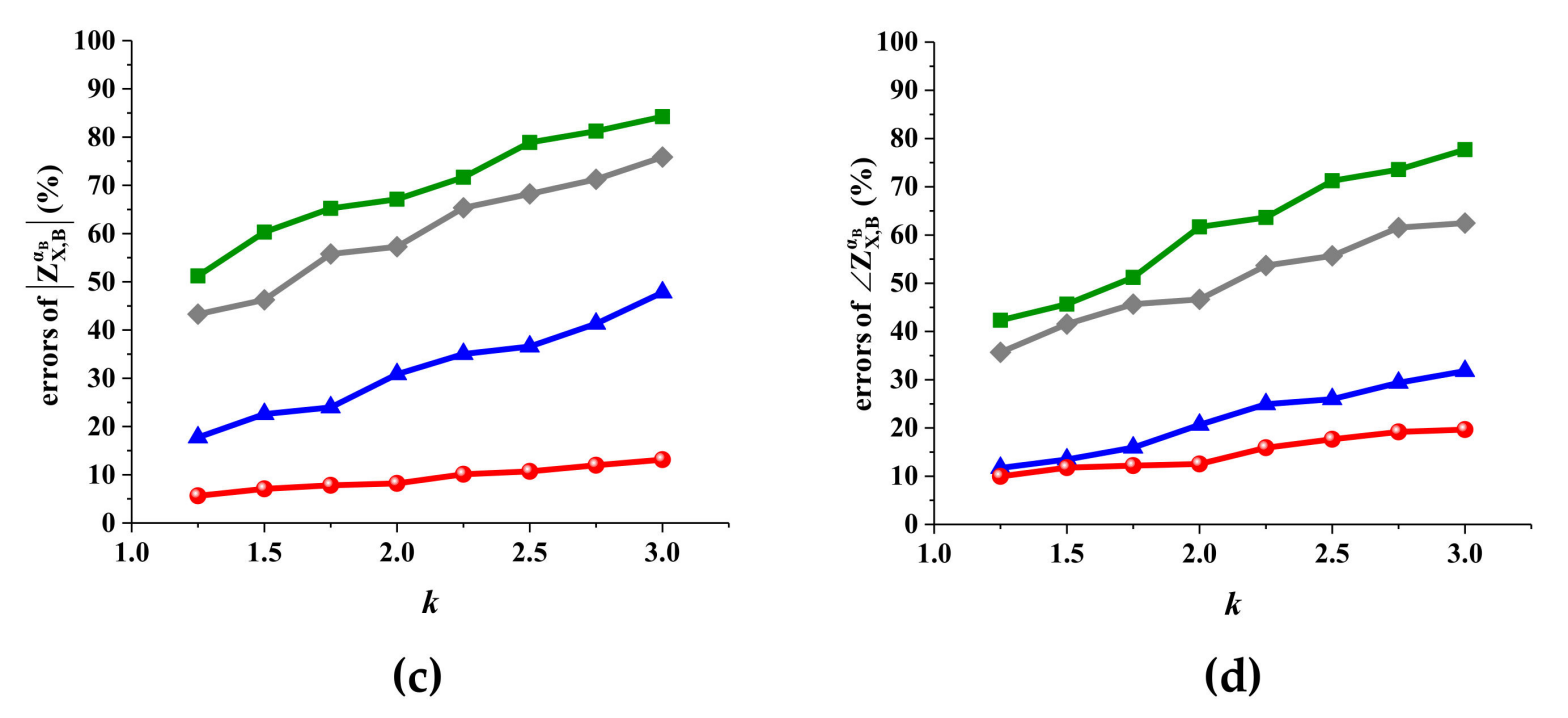
Effects of the background harmonics: (**a**) errors of $\left|Z_{X,A}^{\alpha_{A}}\right|$; (**b**) errors of $∠Z_{X,A}^{\alpha_{A}}$; (**c**) errors of $\left|Z_{X,B}^{\alpha_{B}}\right|$; (**d**) errors of $∠Z_{X,B}^{\alpha_{B}}$.

We further analyze the effects of the background harmonics on the calculation accuracy by setting ${\dot{U}}_{0}=k{\dot{U}}_{0}$. As the coefficient *k* increases, the background harmonics become more unstable. The calculation results of these four methods are shown in Figure 13. The calculation errors of the lease squares and multiple linear regression methods become terrible with the increasing of *k*. Although the results of the traditional CICA are modified compared with the former two methods, the errors still increase rapidly as the background harmonics increase. By contrast, the results of the improved CICA always have high accuracy, satisfying engineering requirements. Therefore, it is further verified that the improved CICA has a stronger ability to resist impacts from the background harmonics.

**Figure 14:**
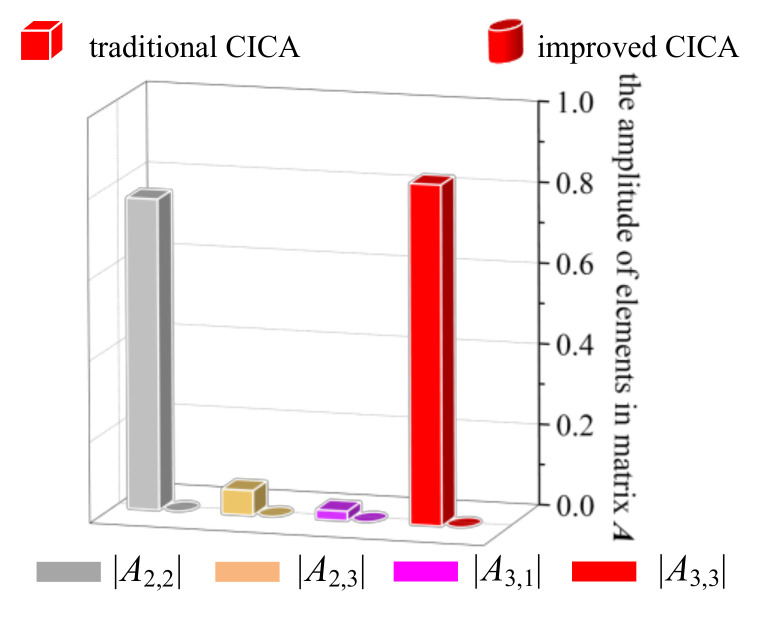
Elements in matrix ***A*** solved from traditional CICA.

To further explore the differences between the traditional and improved CICA, Figure 14 shows the amplitudes of the elements in the solved mixed matrix ***A***. In the improved CICA, the elements *A*_2,2_, *A*_2,3_, *A*_3,1_, and *A*_3,3_ are exactly equal to 0. However, in the traditional CICA, these elements do not exactly equal their theoretical values, which decrease the calculation accuracy.

In addition, real-time implementation is important for an algorithm. Since the traditional CICA is derived from a fast fixed-point algorithm [8], it already has a high execution speed. Further, in the improved CICA, the optimization dimension is decreased based on the sparseness of the matrix ***A***. Thus, the execution time is further reduced. For the whole 10 minutes data, the execution time of the improved CICA is just 0.43 seconds. Thus, with such a high execution speed, the proposed method can be used to evaluate the harmonic contributions in real time.

## 6. Field Case Verifications

**Figure 15:**
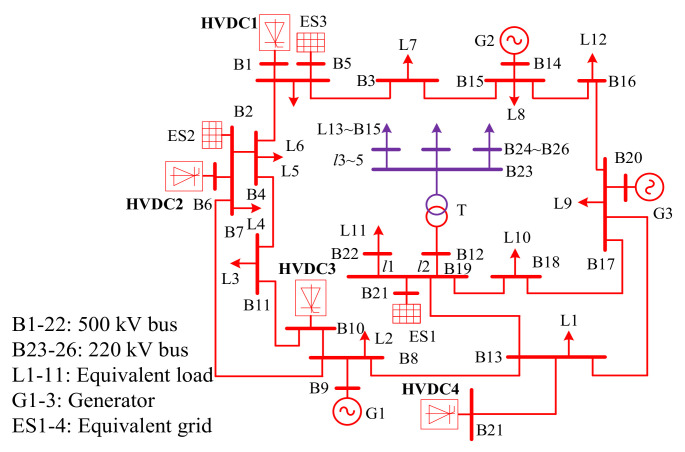
The topology of a multi-infeed HVDC system.

**Figure 16:**
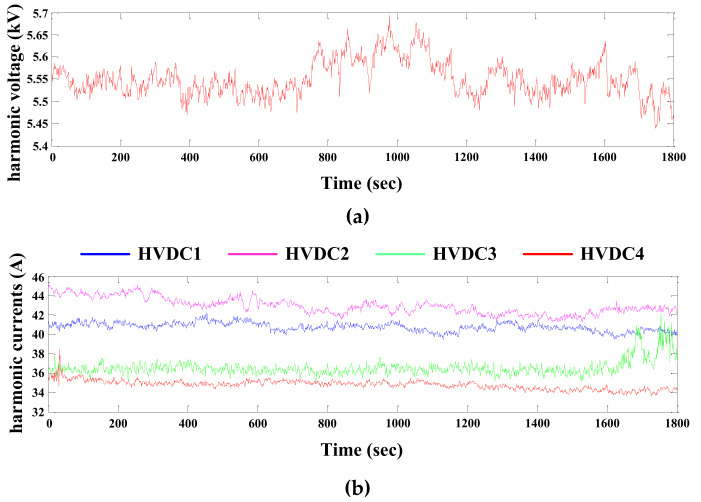
The measured 11th harmonic data: (**a**) 11th harmonic voltages measured at the concerned bus; (**b**) 11th harmonic currents measured at each HVDC.

The power grid for an actual multi-infeed HVDC system shown in Figure 15 is used to further verify the validity of the proposed method under the situation of asynchronous measurements. In this power system, converter stations, as the high-power harmonic sources in a high-voltage level, inject a lot of characteristic harmonics into the power system, which worsen the harmonic distortion in some areas of the grid [32,33]. Bus B23 with high 11th harmonic voltage content exceeding the Chinese standard limits is set as the concerned bus, while the four HVDC systems are the main suspicious harmonic sources. After applying the DFT analysis for the measured data, the 11th harmonic voltages at bus B23 and the currents at each HVDC system are shown in Figure 16. The sampling frequency is 10k Hz and the data resolution is 1 sample per second. Of note, the measured harmonic currents in Figure 16b are asynchronized with the harmonic voltages in Figure 16a. Meanwhile, the corresponding synchronized currents are also measured, and so, the results calculated from the synchronous case can be used as the reference.

**Figure 17:**
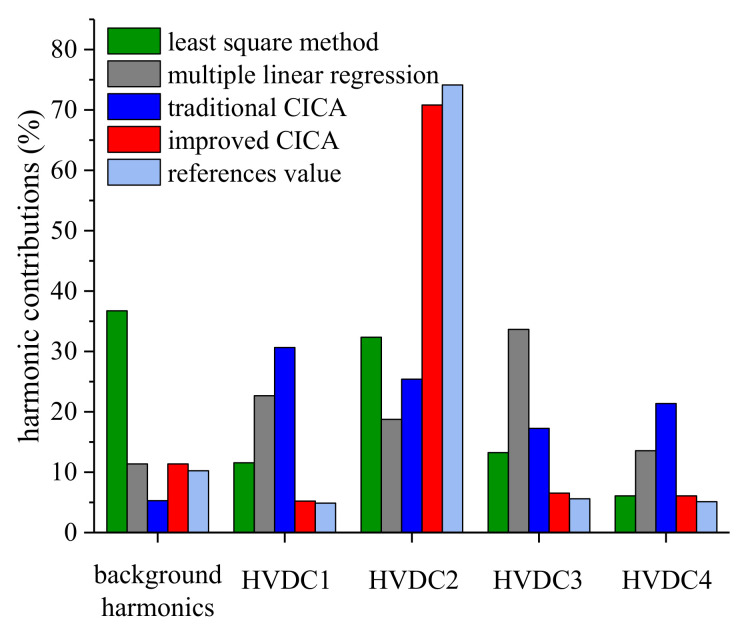
Harmonic contribution of each HVDC.

The harmonic contributions of the four HVDCs calculated from each method with the asynchronous data are shown in Figure 17 and Table 5. Since there are four suspicious harmonic sources, it is hard to satisfy the basic assumption of the least squares method that only one harmonic source fluctuates at a time. Thus, the calculation errors are large. The multiple linear regression method is based on the condition that the relative phase between ${\dot{U}}_{X}$ and each ${\dot{U}}_{i}$ is approximately constant. However, in engineering practice, this phase angle usually varies especially when ${\dot{U}}_{X}$ is generated by multiple harmonic sources. Therefore, the calculation errors of the multiple linear regression method are still large. In addition, for traditional CICA, the large number of suspicious harmonic sources increases the difficulty of signal separation. Thus, the calculation accuracy is low. In comparation, the optimization dimension of the improved CICA is decreased by adopting the sparseness of the matrix ***A***. Hence, the calculation burden is greatly released, and the evaluation accuracy is satisfactory. Therefore, it can be concluded that HVDC2 is the dominant harmonic source for the concerned bus.

**Figure 18:**
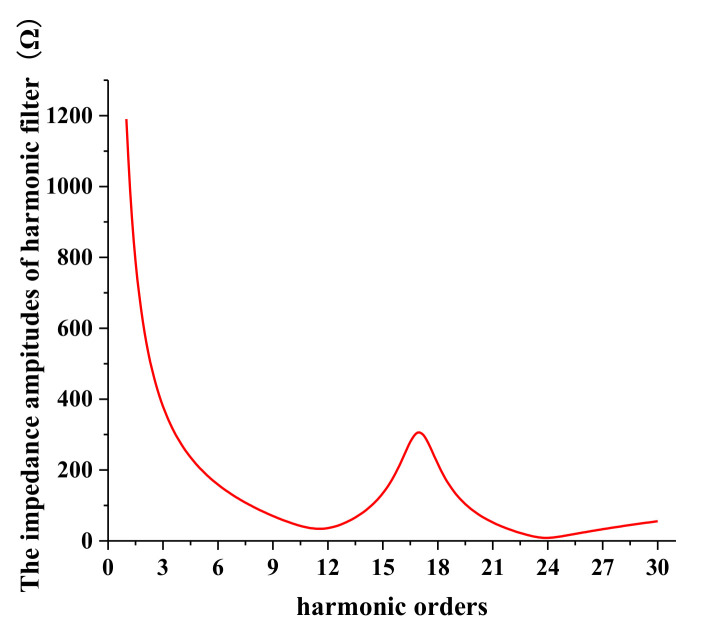
The harmonic impedance property of the double-tuned filter.

To further validate the above conclusion, the contents of the 11th harmonic voltage at the concerned bus are analyzed under different switching modes of harmonic filters at each HVDC. Although harmonic filters are expensive, they are still widely applied in the HVDC converter stations in China. Negative effects on technology and economy will occur without these filters. On the one hand, the harmonic currents injected into the power system far exceed the Chinese standard limits, and the voltages in most buses of the power gird will be seriously distorted. On the other hand, the potential economic loss caused by the characteristic harmonics of HVDCs can be far beyond the cost of the harmonic filters. As a result, filters are necessary for HVDC systems [34]. Figure 18 presents the harmonic impedance property of the double-tuned filters installed at the converter station. The impedance amplitudes corresponding to the 11th, 13th, 23rd, and 25th harmonics are low; thus, the relative characteristic harmonics can be filtered out.

**Figure 19:**
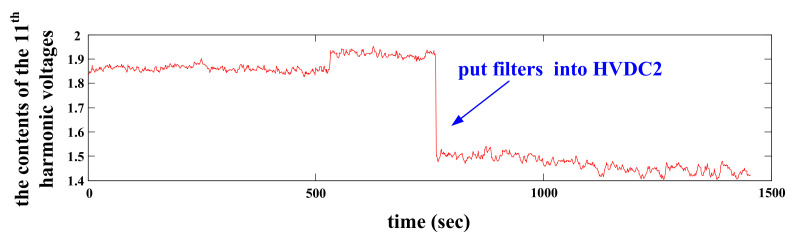
The contents of the 11th harmonic voltage at the concerned bus.

Figure 19 indicates that when more filters are put into HVDC2, the contents of the 11th harmonic voltage at the concerned bus decrease obviously. In contrast, the harmonic mitigation effects are weak after putting more filters into other HVDCs. Consequently, HVDC2 is surely the dominant harmonic source for the concerned bus and the evaluation results from the improved CICA are further validated.

## 7. Conclusions

To evaluate the harmonic contributions accurately and economically, a novel method is proposed in this paper. First, an evaluation model independent of expensive GPS technology is developed. Then, a mixed entropy screening mechanism is designed to select the data segments with stable background harmonics. Last, the optimization dimensions of CICA are greatly reduced by using the sparseness of the mixed matrix, and so the evaluation accuracy is enhanced. The results of simulations and field case studies are summarized as:

(1) For the data segments with fluctuating background harmonics, both $H\left[{\dot{U}}_{X}^{\mathrm{fast}}\right]<H_{m}$ and $\sigma_{H}<4\%$ hold, while the simulation results satisfy $H\left[{\dot{U}}_{X}^{\mathrm{fast}}\right]>H_{m}$ when background harmonics are stable. Thus, the fluctuation degree of background harmonics is successfully assessed by the proposed mixed entropy screening mechanism.
(2) In simulations and filed cases studies, the results of the proposed asynchronous evaluation model are consistent with the harmonic contributions evaluated from the synchronous measurement data, verifying the validity of the proposed GPS-free evaluation model.
(3) Calculation precision of the traditional methods is low especially when the background harmonics are unstable and there are many suspicious harmonic sources. In comparison, owing to the effect of optimization dimensions reduction, the improved CICA always has high evaluation accuracy in simulations and field case studies.

Consequently, compared with the existing studies, the proposed method can evaluate the harmonic contributions accurately even when the background harmonics fluctuate and the number of suspicious harmonic sources is large. Further, the relative evaluation processes no longer rely on expensive GPS. By applying the proposed method, the cost of the harmonic contribution evaluation will greatly decrease. Meanwhile, the accurate evaluation results can be the basis of designing the punishment mechanism for nonlinear customers and identifying the dominated harmonic sources via the proposed method is significant to guide harmonic mitigation.

In future works, we will further apply the evaluation results to release the harmonic pollutions in the power grid.

## Figures and Tables

**Table 1:** Description of the symbols involved in this paper.

Symbol	Description
${\dot{U}}_{X}$	the *h*-th harmonic voltages measured at the concerned bus
${\dot{U}}_{i}$	the harmonic voltages generated by the customer *i* at the concerned bus
${\dot{U}}_{0}$	the background harmonic voltages
$\alpha_{i}$	the unknown relative phase between the synchronous and asynchronous measurements of the customer *i*
$\theta_{i}$	the angle between ${\dot{U}}_{i}$ and ${\dot{U}}_{X}$
$\sigma_{X,i}$	the harmonic contribution of customer *i*
${\dot{I}}_{i}$	the harmonic currents measured at the point of common coupling (PCC) of the customer *i*
${\dot{I}}_{c,i}$	the harmonic currents generated from the customer *i*
${\dot{I}}_{c,i}^{\alpha_{i}}$	the simplified representation of $e^{j\alpha_{i}}{\dot{I}}_{c,i}$
$Z_{c,i}$	the harmonic impedance of the customer *i*
$Z_{u,i}$	the harmonic impedance of the utility side of the customer *i*
$Z_{X,i}$	the transfer impedance between the customer *i* and bus X
$Z_{X,i}^{\alpha_{i}}$	the simplified representation of $e^{-j\alpha_{i}}Z_{X,i}$
superscript “^fast^”	the fast-varying components of a signal
superscript “^^^”	the calculated results
superscript “^T^”	the Hermitian transpose
X	the observed signals in the blind source separation model
A	the mixed matrix in the blind source separation model
S	the source signals in the blind source separation model
Y	the signals separated from the complex independent component analysis (CICA)
$X_{W}$	signal *X* after being whitened
Q	the whitening matrix of the observed signals
Λ	the diagonal matrix of eigenvalues of *E*{*XX*^T^}
Γ	the orthogonal matrix of eigenvalues of *E*{*XX*^T^}
W	the separating matrix
Θ	the inverse of Q
$H\left(s\right)$	the entropy of a signal
$H\left[s\right]$	the mixed entropy of a signal
$\sigma_{H}$	an index to assess the stability of the background harmonics
$H_{m}$	the average value of $H\left[{\dot{U}}_{X}^{\mathrm{fast}}\right]$ for all the data segments
E{⋅}	calculate the mean value of a signal
$std\left\{\cdot \right\}$	calculate the standard deviation of a signal
$J_{G}(\cdot )$	calculate the negative entropy of a signal

**Table 2:** Results of the mixed entropy screening.

Data Segment	Mixed Entropy			$\sigma_{H}\;(\%)$
	$H\left[{\dot{U}}_{X}^{\mathrm{fast}}\right]$	$H\left[{\left({\dot{I}}_{c,A}^{\alpha_{A}}\right)}^{\mathrm{fast}}\right]$	$H\left[{\left({\dot{I}}_{c,B}^{\alpha_{B}}\right)}^{\mathrm{fast}}\right]$	
1	0.815	0.671	0.615	21.461
2	0.801	0.654	0.643	22.477
3	0.734	0.673	0.629	9.064
4	0.792	0.666	0.582	18.919
5	0.691	0.671	0.621	2.981
6	0.799	0.626	0.622	27.636
7	0.705	0.673	0.708	−0.424
8	0.809	0.647	0.668	21.108
9	0.738	0.629	0.636	16.038
10	0.793	0.639	0.612	24.1
mean value	0.768	0.655	0.634	16.34

**Table 3:** Calculation errors of the transfer impedances.

Method	Calculation Errors of the Transfer Impedances (%)			
	$\left|Z_{X,A}^{\alpha_{A}}\right|$	$∠Z_{X,A}^{\alpha_{A}}$	$\left|Z_{X,B}^{\alpha_{B}}\right|$	$∠Z_{X,B}^{\alpha_{B}}$
least squares method	53.68	47.16	45.17	37.36
multiple linear regression	38.91	41.57	46.72	31.41
traditional CICA	19.96	21.43	23.37	22.57
improved CICA	8.73	8.46	6.71	7.38

**Table 4:** Harmonic contributions for each harmonic source.

Method	Harmonic Contributions (%)		
	Harmonic Source A	Harmonic Source B	Background Harmonics
least squares method	23.35	61.79	14.86
multiple linear regression	32.78	48.64	18.58
traditional CICA	57.34	31.47	11.19
improved CICA	52.28	19.16	28.56
reference value	48.32	21.47	30.21

**Table 5:** Harmonic contributions calculated by each method.

Method	Harmonic Contributions (%)				
	Background Harmonics	HVDC1	HVDC2	HDVC3	HVDC4
least squares method	36.74	11.56	32.36	13.25	6.09
multiple linear regression	11.37	22.68	18.74	33.66	13.55
traditional CICA	5.28	30.65	25.42	17.27	21.38
improved CICA	11.37	5.21	70.82	6.53	6.07
reference value	10.24	4.88	74.14	5.61	5.13
